# LRScaf: improving draft genomes using long noisy reads

**DOI:** 10.1186/s12864-019-6337-2

**Published:** 2019-12-09

**Authors:** Mao Qin, Shigang Wu, Alun Li, Fengli Zhao, Hu Feng, Lulu Ding, Jue Ruan

**Affiliations:** Guangdong Laboratory for Lingnan Modern Agriculture, Genome Analysis Laboratory of the Ministry of Agriculture, Agricultural Genomics Institute at Shenzhen, Chinese Academy of Agricultural Sciences, No. 7, Pengfei Road, Dapeng District, Shenzhen, 518120 Guangdong China

**Keywords:** LRScaf, Scaffolding algorithm, Third generation sequencing technologies, PacBio, Nanopore

## Abstract

**Background:**

The advent of third-generation sequencing (TGS) technologies opens the door to improve genome assembly. Long reads are promising for enhancing the quality of fragmented draft assemblies constructed from next-generation sequencing (NGS) technologies. To date, a few algorithms that are capable of improving draft assemblies have released. There are SSPACE-LongRead, OPERA-LG, SMIS, npScarf, DBG2OLC, Unicycler, and LINKS. Hybrid assembly on large genomes remains challenging, however.

**Results:**

We develop a scalable and computationally efficient scaffolder, Long Reads Scaffolder (LRScaf, https://github.com/shingocat/lrscaf), that is capable of significantly boosting assembly contiguity using long reads. In this study, we summarise a comprehensive performance assessment for state-of-the-art scaffolders and LRScaf on seven organisms, i.e., *E. coli*, *S. cerevisiae*, *A. thaliana*, *O. sativa*, *S. pennellii*, *Z. mays*, and *H. sapiens*. LRScaf significantly improves the contiguity of draft assemblies, e.g., increasing the NGA50 value of CHM1 from 127.1 kbp to 9.4 Mbp using 20-fold coverage PacBio dataset and the NGA50 value of NA12878 from 115.3 kbp to 12.9 Mbp using 35-fold coverage Nanopore dataset. Besides, LRScaf generates the best contiguous NGA50 on *A. thaliana*, *S. pennellii*, *Z. mays*, and *H. sapiens*. Moreover, LRScaf has the shortest run time compared with other scaffolders, and the peak RAM of LRScaf remains practical for large genomes (e.g., 20.3 and 62.6 GB on CHM1 and NA12878, respectively).

**Conclusions:**

The new algorithm, LRScaf, yields the best or, at least, moderate scaffold contiguity and accuracy in the shortest run time compared with other scaffolding algorithms. Furthermore, LRScaf provides a cost-effective way to improve contiguity of draft assemblies on large genomes.

## Background

With the advent of next-generation sequencing (NGS) technologies, the genomics community has made significant contributions to de novo genome assembly. Despite that many studies and tools are aimed at reconstructing NGS data into complete de novo genomes, this goal is challenging to achieve because of an intrinsic limitation of NGS data, i.e., read lengths are shorter than most of the repetitive sequences [1]. The existence of repeats makes it challenging to reconstruct a complete genome instead of generating lots of contiguous sequences (contigs) even when the sequencing coverage is high [2]. Thus, attention has focused on the so-called genomic scaffolding procedure, which aims at reducing the number of contigs by using fragments of moderate lengths whose ends are sequenced (double-barreled data) [3, 4]. Nevertheless, long repetitive sequences still limit genomic assembly.

As the development of third-generation sequencing (TGS) technologies, it sheds light on different alternatives to solve genome assembly problems by offering very long reads. For example, the single-molecule, real-time (SMRT) sequencing technology of Pacific Biosciences® (PacBio) delivers read lengths of up to 50 kbp [5], and the nanopore sequencing technology of Oxford Nanopore Technologies® (Nanopore) yields read lengths that are greater than 800 kbp [6]. Also, the Hi-C data provides much longer-range linking information than other technologies (such as mate pairs, optical maps, linked reads) [7]. With the TGS long reads and the Hi-C data on de novo assembly, it is possible to reconstruct genome into complete chromosome arms [8, 9]. However, these long reads suffer from high sequencing error rates, which necessitate high coverage during the de novo assembly [10]. Also, TGS technologies have a higher cost per base than NGS methods, and the Hi-C data would produce small inversions within the scaffolds when the draft assemblies are not with good quality and contiguity [11]. On a large-scale assembly project (such as the ruminant project [12]), a more reasonable and cost-effective way is to improve the contiguity of draft assemblies constructed by NGS data with low coverage long reads [7, 13].

The process of genome assembly typically divides into two major steps. The first step is to piece-by-piece overlap reads into contigs. This step commonly performs using *de Bruijn* or overlap graphs [1]. The second step is to assemble scaffolds that consist of ordered the oriented contigs with estimated distances between them. Scaffolding, which was first introduced by Huson [3], is a critical part of the genome assembly process, especially for NGS data. Scaffolding is an active research area because of NP-hard complexity [14]. By using paired-end and/or mate-pair reads, a number of standalone scaffolders, e.g., Bambus [4], MIP [15], Opera [16], SCARPA [17], SOPRA [18], SSPACE [19], BESST [20], and BOSS [21] have been developed. A recent comprehensive evaluation showed that scaffolding remains computationally intractable and requires larger insert-size and higher quality pair read libraries than what is presently available [22]. As TGS technologies are likely to offer longer reads than the lengths of the most common repeats, these technologies are capable of drastically reducing and overcoming the complexity caused by repeats. Considering the pros and cons of NGS and TGS data, a hybrid assembly approach that assembles draft genomes using TGS data was proposed [23]. The core strategy of this approach is: 1) long reads are mapped onto the contigs using a long-read mapper (e.g., BLASR [24] or minimap [25]); 2) alignment information is examined, and long reads that span more than one contig are identified and their linking relationship is stored in a data structure; 3) the last step is to clean up the structure by removing redundant and error-prone links, calculate distances between contigs, and build scaffolds using linking information.

AHA was the first standalone scaffolder basing on the hybrid-assembly strategy, and this algorithm was part of the SMRT analysis software suite [23]. As AHA is designed for small genomes and has limitations on the input data, it is not suitable for large genomes. To ensure that scaffolds are as contiguous as possible, AHA performs six iterations by default, thus increasing the run time. For comparison, SSPACE-LongRead [26] produces final scaffolds in a single iteration and, therefore, has a significantly shorter run time than AHA. Nevertheless, SSPACE-LongRead has somewhat lower assembly accuracy than AHA. Despite being designed for eukaryotic genomes, the run time of SSPACE-LongRead is unpractical on large genomes. LINKS [27] opens a new door to building linking information between contigs. The algorithm uses the long interval nucleotide K-mer without computational alignment and a reads-correction step. The memory usage of LINKS is noteworthy. OPERA-LG [28] provides an exact algorithm for large and repeat-rich genomes. It requires significant mate-pair information to constrain the scaffold graph and yield an optimised result. OPERA-LG is not directly designed for TGS data. To construct scaffold edges and link contigs into scaffolds, OPERA-LG needs to simulate and group mate-pair relationship information from long reads. Recently, scaffolding algorithms, such as SMIS (http://www.sanger.ac.uk/science/tools/smis), npScarf [29], DBG2OLC [30], and Unicycler [31], incorporate the hybrid-assembly strategy. However, these tools have not been thoroughly assessed for different genome sizes, especially large genomes.

Here we present a Long Reads Scaffolder (LRScaf) that improves draft genomes using TGS data. The input to LRScaf is given by a set of contigs and their alignments over PacBio or Nanopore long reads. We compare our algorithm with state-of-the-art tools on datasets for seven species (i.e., *Escherichia coli*, *Saccharomyces cerevisiae*, *Arabidopsis thaliana*, *Oryza sativa*, *Solanum pennellii*, *Zea mays*, and *Homo sapiens*). All the tested methods improve the contiguity of pre-assembled genomes. LRScaf yields the best assembly metrics and contiguity for the pre-assembled genomes on *E. coli*, *S. cerevisiae*, *A. thaliana*, *S. pennellii*, *Z. mays*, and *H. sapiens*. More importantly, our method consistently returns high-quality scaffolds and has the shortest run time. LRScaf significantly improves the contiguity of human draft assemblies, increasing the NGA50 value of CHM1 from 127.1 kbp to 9.4 Mbp with 20-fold coverage PacBio dataset and the NGA50 value of NA12878 from 115.3 kbp to 12.9 Mbp with 35-fold coverage Nanopore dataset. Thus, we show that LRScaf is a promising tool for improving draft assemblies in a computationally cost-effective way.

## Implementation

### Experimental data

The present study performs on two small genomes (*E. coli* and *S. cerevisiae*), three medium genomes (*A. thaliana*, *O. sativa*, and *S. pennellii*), and two large genomes (*Z. mays* and *H. sapiens*). All the tested data of the seven species are collected from published datasets except the Illumina dataset of *O. sativa,* which is sequenced in this study (Table 1). For the PacBio long reads datasets, we select the first 20-fold coverage of each PacBio dataset to assess all the scaffolders comprehensively. To test all the scaffolders’ performances on the lower depths, we use three different coverages, i.e., 1, 5, and 10 -fold for the two small genomes and 1, 5, and 15 -fold for *H. sapiens* (NA12878). To assess scaffolder performances for different median read lengths, we use three different medians (8, 18, and 26 kbp) of 10-fold coverage on the *E. coli* PacBio datasets. The coverage of the Nanopore long reads datasets is not too high, and, therefore, we use all of the long reads data from these datasets to assess scaffolder performances.

**Table 1:** Descriptive statistics of datasets for the experiment

Species	Type	Total bases (bp)	Coverage	Median (bp)	Longest (bp)	Source
*E. coli*	Illumina	350,000,031	70.0 X	100	100	ERA000206
	Illumina ^a^	256,927,500	54.9 X	300	300	SRR826442; SRR826444; SRR826446; SRR826450
	PacBio	93,994,356	20.1 X	8712	41,331	SRX669475; SRX533603
	Nanopore ^(2D) a^	136,895,083	29.2 X	6153	43,600	http://gigadb.org/dataset/100102
	Nanopore ^(Full) b^	21,972,483	4.7 X	5743	47,422	ERX708228
	Nanopore ^(All) b^	158,867,566	34.0 X	6086	47,422	ERX708228
	Nanopore ^(Raw) b^	311,558,723	66.5 X	3557	94,116	ERX708228
*S. cerevisiae*	Illumina	1,268,786,706	105.1 X	202	202	SRR527545; SRR527546
	PacBio	249,319,042	20.7 X	4554	27,575	SRX533604
	Nanopore ^(Nanocorr) b^	526,588,732	43.6 X	5512	72,879	SRP055987
	Nanopore ^(Raw) b^	2,392,848,698	198.2 X	5059	191,145	SRP055987
*A. thaliana* ^(KBS-Mac-74)^	Illumina	8,420,975,500	70.3 X	250	250	ERR2173372
	Nanopore	3,421,779,258	28.6 X	7543	269,087	ERR2173373
*A. thaliana* ^(ler-0)^	Illumina ^a^	6,919,422,000	59.0 X	300	300	http://schatzlab.cshl.edu/data/ectools/
	PacBio ^a^	2,400,246,920	20.5 X	15,357	41,753	http://schatzlab.cshl.edu/data/ectools/
*O. sativa*	Illumina	43,519,132,800	111.5 X	150	150	PRJNA515358 ^c^
	PacBio	7,999,992,602	20.5 X	4117	50,493	PRJNA318714
*S. pennellii*	Illumina	39,007,839,296	42.6 X	311	311	PRJEB19787
	Nanopore	27,483,806,911	30.0 X	13,061	15,387	PRJEB19787
*Z. mays*	PacBio	49,999,992,839	23.7 X	1347	17,784	PRJNA10769
*H. sapiens* ^(CHM1)^	PacBio	59,999,995,767	20.0 X	1569	208,628	SAMN02744161
*H. sapiens* ^(NA12878)^	Nanopore	114,380,310,980	35.0 X	4569	1,537,349	PRJEB23027

*Note*: ^a^ refers to DBG2OLC dataset; ^b^ refers to LINKS dataset; ^c^ the dataset was sequenced in this study

### Producing draft assemblies

For the two small genomes, the draft assemblies are constructed by SOAPdenovo2 [32] and SPAdes [33]. To assess the performances between LINKS and the other scaffolders on the Nanopore datasets, the draft assemblies which are published on LINKS are also included (Table 2). The NGS assemblers, i.e., DISCOVAR [34], MaSuRCA [35], Platanus [36], SOAPdenovo2, and SparseAssembler [37] are used to generate the draft assemblies for *A. thaliana* (KBS-Mac-74), *O. sativa*, and *S. pennellii*. To compare with DBG2OLC, we generate the draft assemblies for *E. coli* and *A. thaliana* (ler-0) using SparseAssembler. The best parameters for these NGS assemblers are determined by taking assembly size and contiguity into account. For the *Z. mays*, *H. sapiens* (CHM1), and *H. sapiens* (NA12878), the released assemblies are used because the computational resources required to determine optimised assembly parameters for these species exceed our platform capacity.

**Table 2:** The summary of draft assemblies of *E. coli*, *S. cerevisiae*, *A. thaliana*, *O. sativa*, *S. pennellii*, *Z. mays* and *H. sapiens*

Species	Method/Source	Sum	NG50	NGA50	Longest	Misassemblies (#)	BUSCO (Complete)
*E. coli*	SOAPdenovo2	4.6 Mbp	25.2 kbp	25.2 kbp	91.7 kbp	0	97.3%
	SPAdes	4.6 Mbp	112.4 kbp	105.6 kbp	265.2 kbp	2	98.6%
	ABySS ^a^	5.2 Mbp	179.7 kbp	146.9 kbp	358.7 kbp	5	98.6%
	SparseAssembler ^b^	4.4 Mbp	3.0 kbp	3.0 kbp	14.9 kbp	2	64.9%
*S. cerevisiae*	SOAPdenovo2	12.1 Mbp	18.7 kbp	18.6 kbp	146.7 kbp	3	96.2%
	SPAdes	11.8 Mbp	104.1 kbp	85.7 kbp	451.4 kbp	22	97.2%
	Celera Assembly ^a^	14.9 Mbp	58.8 kbp	54.7 kbp	257.3 kbp	19	98.7%
*A. thaliana* ^(KBS-Mac-74)^	DISCOVAR	117.9 Mbp	323.0 kbp	314.6 kbp	2.5 Mbp	67	98.5%
	MaSuRCA	119. 5 Mbp	413.2 kbp	356.5 kbp	1.7 Mbp	145	98.3%
	Platanus	113.0 Mbp	145.5 kbp	143.7 kbp	800.8 kbp	31	98.3%
	SOAPdenovo2	115.1 Mbp	236.7 kbp	227.0 kbp	1.5 Mbp	39	98.3%
	SparseAssembler	93.0 Mbp	12.8 kbp	12.7 kbp	114.5 kbp	1	94.7%
*A. thaliana* ^(ler-0)^	SparseAssembler ^b^	74.7 Mbp	4.4 kbp	4.2 kbp	35.8 kbp	90	74.6%
*O. sativa*	DISCOVAR	313.8 Mbp	27.1 kbp	23.6 kbp	262.5 kbp	1343	96.9%
	MaSuRCA	339.2 Mbp	30.6 kbp	29.1 kbp	219.4 kbp	1288	96.7%
	Platanus	307.9 Mbp	16.8 kbp	16.6 kbp	154.3 kbp	367	95.6%
	SOAPdenovo2	301.2 Mbp	18.5 kbp	18.3 kbp	207.7 kbp	91	97.1%
	SparseAssembler	155.3 Mbp	–	–	43.0 kbp	2	85.8%
*S. pennellii*	DISCOVAR	851.9 Mbp	66.4 kbp	59.6 kbp	1.3 Mbp	4235	94.2%
	MaSuRCA	884.2 Mbp	61.3 kbp	54.9 kbp	617.2 Mbp	6621	94.9%
	Platanus	641.3 Mbp	15.4 kbp	15.2 kbp	270.1 kbp	115	91.7%
	SOAPdenovo2	768.5 Mbp	28.2 kbp	26.8 kbp	323.3 kbp	632	92.6%
	SparseAssembler	305.2 Mbp	–	–	51.1 kbp	11	76.5%
*Z. mays*	PhredPhrap+ABySS (GCA_000005005.5)	2.0 Gbp	40.0 kbp	36.2 kbp	849.5 kbp	15,133	91.9%
*H. sapiens* ^(CHM1)^	SRPRISM+ARGO (GCF_000306695.2)	2.8 Gbp	127.5 kbp	127.1 kbp	1.0 Mbp	106	80.3%
*H. sapiens* ^(NA12878)^	DISCOVAR (GCA_001517065.1)	2.8 Gbp	115.7 kbp	115.3 kbp	961.2 kbp	336	83.7%

*Note*: ^a^ refers to LINKS dataset; ^b^ refers to DBG2OLC dataset; “-”: Not available

### Alignment validation and repeat identification

**Fig. 1:**
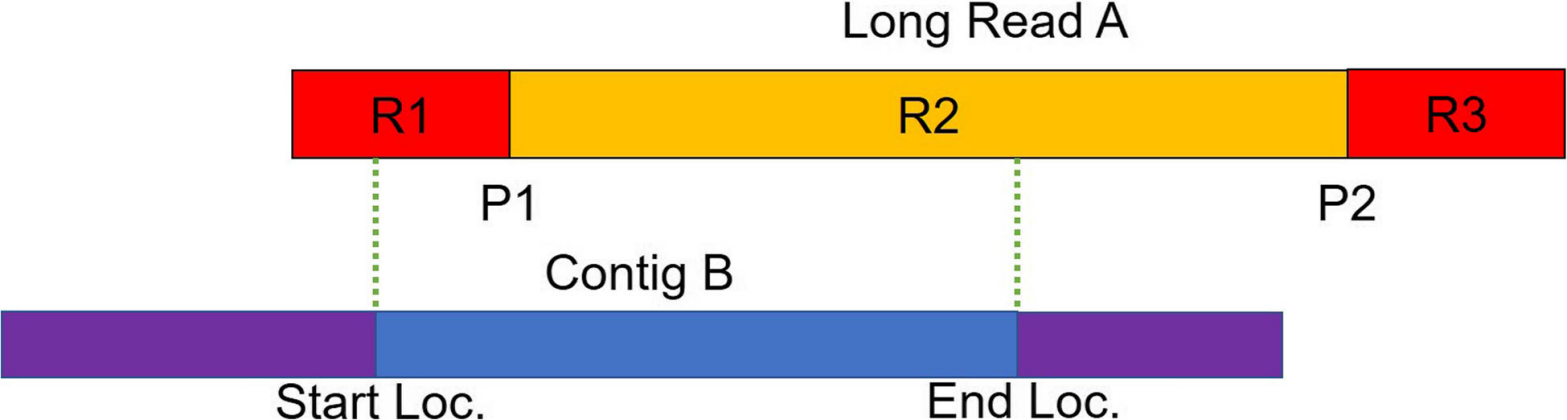
A validating model of alignment. The P1 and P2 are the two points for breaking a long read into three regions (R1, R2, and R3).

LRScaf is designed to separate the mapping and scaffolding procedures and supports BLASR and minimap (Version 1 and 2). The high error rate is a severe disadvantage of TGS long reads. Thus, a significant fraction of the alignments is incorrect and needs to be filtered out. We develop a validation model to validate each alignment (Fig. 1). The model partitions each long read into three regions (R1, R2, and R3) that are separated by two points (P1 and P2). There are six different combination sets in *R* if the alignment start (S) and end (E) loci of the contig are considered, i.e., *R* ∈ {(*S in R*1, *E in R*1),  (*S in R*1, *E in R*2),  (*S in R*1, *E in R*3),  (*S in R*2, *E in R*2),  (*S in R*2, *E in R*3),  (*S in R*3, *E in R*3)}. We also define the distal length of a contig to the start or end alignment loci as the over-hang length of the contig. Taking both the alignment region and the over-hang length into account, the valid alignment satisfies: 1) (S in R1, E in R1) with the right over-hang length not exceeding the constraints; 2) (S in R1, E in R2) with the right over-hang length not exceeding the constraints; 3) (S in R2, E in R2) with the over-hang length of two ends not exceeding the constraints; 4) (S in R2, E in R3) with the left over-hang length not exceeding the constraints; 5) (S in R3, E in R3) with the left over-hang length not exceeding the constraints. An alignment is filtered out if a long read is entirely covered by a contig (S in R1, E in R3), i.e., the contig contains the long read. After this procedure, the remaining alignments are considered to be valid for the scaffolding procedure.

Repetitive sequences complicate genome assembly. Thus, such sequences are masked in our approach. First, we identify and remove repeats by the coverage of reads based on the uniform coverage of TGS data. In the calculation of reads coverage, long reads that covered the entire contig are counted. Then we compute the mean coverage and the standard deviation among the set of contigs. Any contig coverage is higher than the threshold coverage which is *μ*_*cov*_ + 3 × *s*. *d*._*cov*_ by default. It is considered to be a repeat, and the corresponding contig is removed from the next step of the analysis.

### Construction of links and edges

**Fig. 2:**
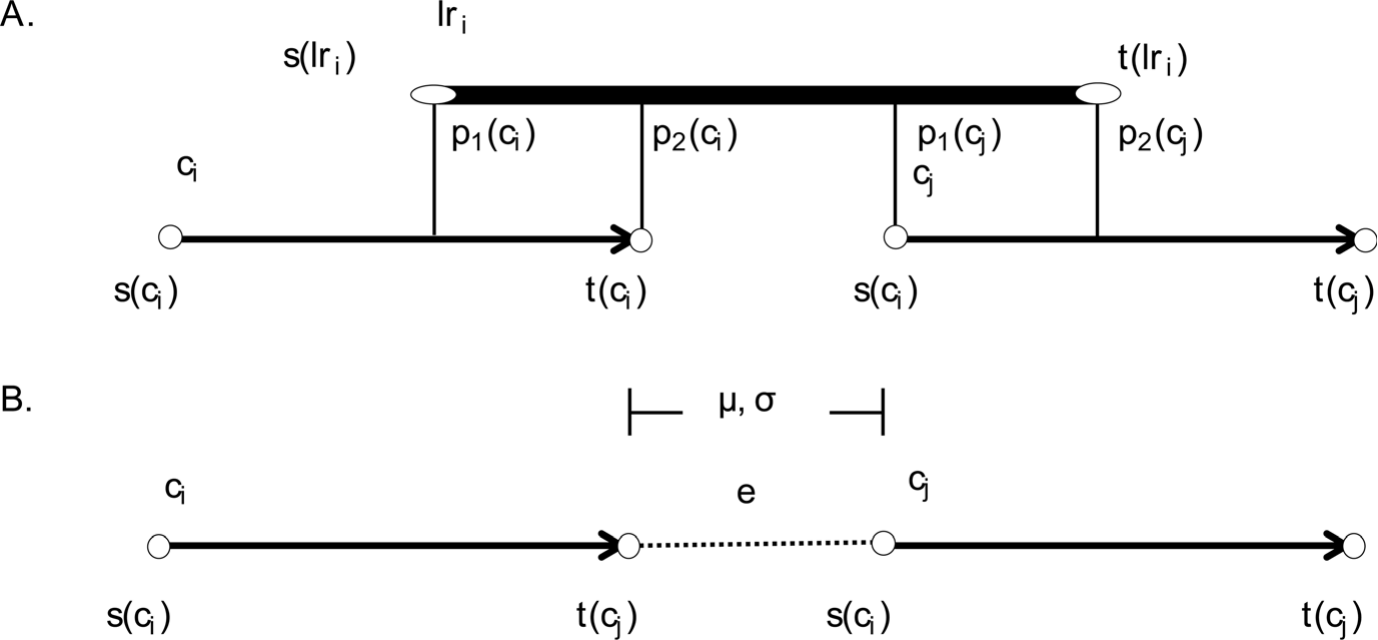
The construction of a link using a long read *lri* and two contigs *ci* and *cj*. **a** A basic schematic for a long read building link between contigs. **b** The distance distribution of links.

A long read may have multiple mappings because of repeats and high sequencing error rates. Figure 2 shows how links are built between contigs from the validated alignments. This process has constraints on orientation and distance. Four strand combination sets, *S*, are used between contigs to constrain orientation, i.e.*, S* ∈ {*s*_1_ : (+, +),  *s*_2_ : (+, −), *s*_3_ : (−, +),  *s*_4_ : (−, −)}. We define the orientation between contigs as *O*(*c*_*i*_, *c*_*j*_) =  *max* (*s*). The probability that the internal distance, *e*, between two contigs lies outside the range [*μ*_*is*_ − 3 × *σ*_*is*_, *μ*_*is*_ + 3 × *σ*_*is*_] is less than 5% because *e* approximately follows a normal distribution *N*(*μ*_*is*_, *σ*_*is*_). When *e* is out of the range [*μ*_*is*_ − 3 × *σ*_*is*_, *μ*_*is*_ + 3 × *σ*_*is*_], it is considered to be abnormal, and the linking information is removed. Any long reads linking a contig to itself at different loci are also removed. After validating that the two constraints on links between contigs are fulfilled, we introduce an edge to represent a bundle of links that join two contigs using quadruple parameters $E\left({c}_i,{c}_j\right)=\left(n,\overline{\mu_{is}}, \overline{\sigma_{is}},o\right)$. Here, *n* is the number of remaining links considering as the weight of the edge, $\overline{\mu_{is}\ }$ is the mean internal distance for the remaining links, $\overline{\sigma_{is}}$ is the standard deviation of the internal distances for the remaining links, and *o* is the orientation strand between contigs.

### Graph generation and simplification

In this step, LRScaf constructs a scaffold graph *G*(*V*, *E*) similar to the string graph formulation. The vertex set, *V*, represents the end of the contigs, and the edge set, *E*, represents the linkage implied by the long reads between ends of two contigs with weight and orientation functions assigned to each edge. Their ID annotates the ends of each contig with a forward strand (+). Using this node concept, there are 4 types of edges in the graph, i.e., (+, +) joining the forward strands of both contigs, (+, −) joining the forward strand of the first contig with the reverse strand of the second contig, (−, +) joining the reverse strand of the first contig with the forward strand of the second contig, and (−, −) joining the reverse strands of both contigs. After the edges-construction step, we account for the majority of the sequencing errors by removing all the edges that have a lower number of long reads than the threshold value. Once the edges have been cleaned and filtered, we construct an assembly graph *G*. We only add an edge to *G* if neither of the two nodes comprising the edge is present in *G*. In some cases, *G* contains some edges of transitive reduction, error-prone and tips. After deleting these edges, we obtain the final scaffold graph, which we use for further analysis.

### Scaffolding contigs into scaffolds

**Fig. 3:**
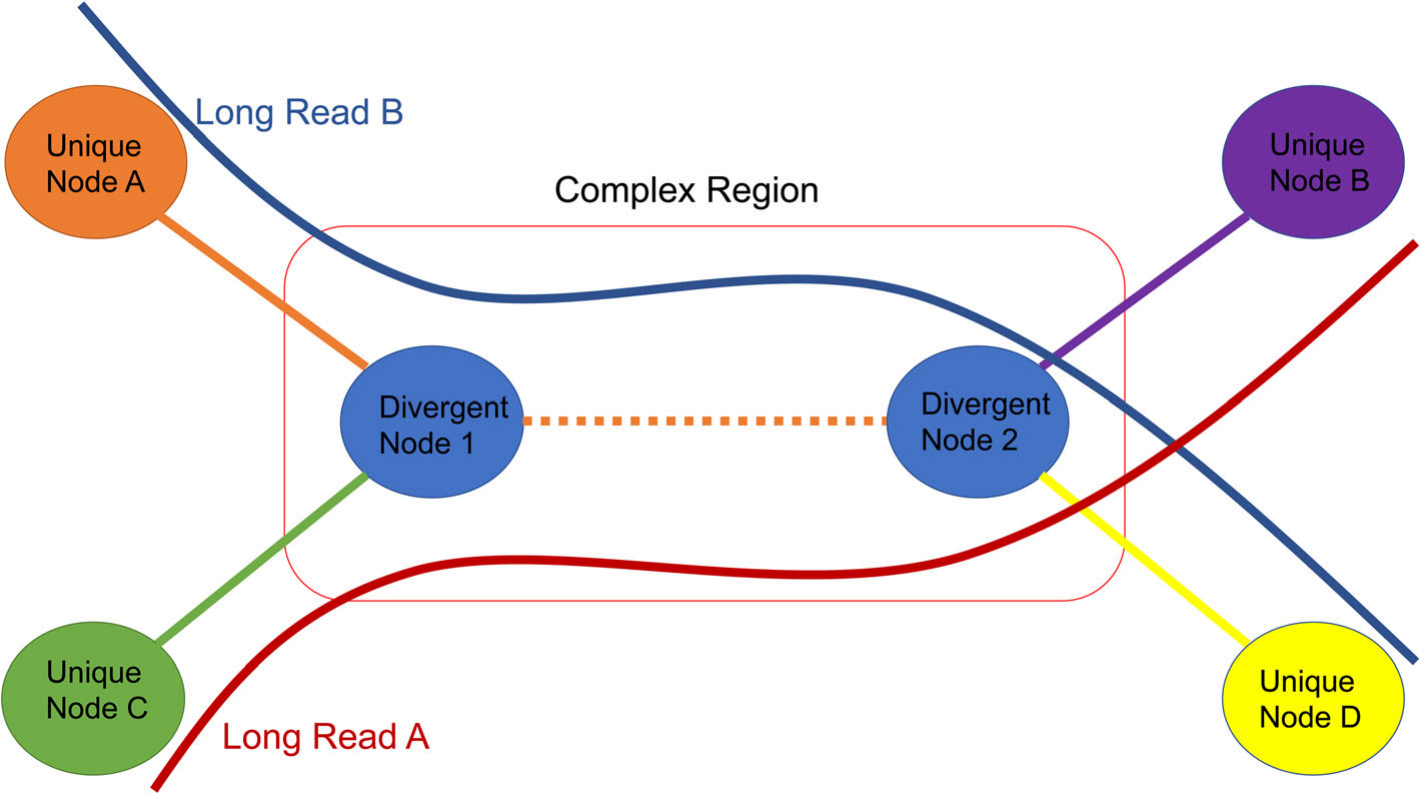
The schematic illustration for travelling the complex region

After the repeat identification and the graph simplification steps, most of the contigs are connected in linear stretches on the assembly graph. There are, however, some complex regions that required addition manipulation. We refer to a contig as a divergent node if it links more than two nodes in the graph (Fig. 3). LRScaf searches for unique nodes at the end of this complex region and steps through this region for as long as there are long reads that join two unique nodes. If a divergent node is reached, LRScaf stops travelling the graph in the forward direction and switches to the reverse direction. Similarly, the search along the reverse direction of the graph stops at the end of a linear stretch or a divergent node. The process is then repeated using an unvisited node as the starting node. The procedure ends after traversing all the unvisited and unique nodes in the graph, thus exposing all linear paths. Finally, the gap-size between contigs is calculated. If the gap-size value is negative, the contigs are merged into a combined contig, and if the value is positive, a gap is inserted between the contigs (a gap is represented by one or more undefined ‘N’ nucleotides, depending on gap-size).

### The parameters of LRScaf

LRScaf has three sections of parameters, i.e., 1) a file-related section, 2) an alignment-validation section, and 3) a performance-related section. The parameter settings can be provided via the program’s command line or through an XML configuration input file. In the file-related section, input parameters are required for the alignment file, draft assemblies, and output path. In the alignment-validation section, there are six mandatory parameters for the alignment-validation model. These are min_overlap_length, min_overlap_ratio, max_overhang_length, max_overhang_ratio, max_end_length, and max_end_ratio. Whereas loosening these parameters improves assembly contiguity, the number of misassemblies increases. In the performance-related section, there are 7 parameters, i.e., min_contig_length, identity, min_supported_links, repeat_mask and tip_length, iqr_time, and process. It will sometimes be necessary to change the values of these parameters based on data. If, for example, long-read coverage is low and assembly contiguity is the main priority, reducing the values of identity and min_supported_links could significantly improve the assembly contiguity. Besides, masking repeats could decrease the number of divergent nodes in the scaffolding graph and generate more contiguous scaffolds.

## Results and discussion

We perform in-depth analysis on seven species (Table 1), i.e., *E. coli*, *S. cerevisiae*, *A. thaliana*, *O. sativa*, *S. pennellii*, *Z. mays*, and *H. sapiens*, to test and compare the performances of LRScaf with that of SMIS, npScarf, DBG2OLC, Unicycler, SSPACE-LongRead, LINKS, and OPERA-LG. For the two small genomes (*E. coli* and *S. cerevisiae*), we assess the performances of all the scaffolders on four different depths and three different medians of long reads (Additional file 1 Supplementary Note). For the three medium genomes (*A. thaliana*, *O. sativa*, and *S. pennellii*) and two large genomes (*Z. mays* and *H. sapiens*), because Unicycler and npScarf are for small genomes and the memory requirement of LINKS exceeds our system’s capacity, we do not perform the benchmarks of these three scaffolders. Platanus and SparseAssembler are the recommended NGS de novo assemblers for DBG2OLC [30, 38]. We do not perform the comparisons for DBG2OLC on the draft assemblies generated by other NGS assemblers. The run time for SSPACE-LongRead exceeds the one-month time limit on the large genomes (*H. sapiens*). We exclude the benchmarks of SSPACE-LongRead on *H. sapiens*. Using DBG2OLC datasets on *E. coli* and *A. thaliana*, we perform the comparisons between DBG2OLC and LRScaf (Additional file 1 Supplementary Note). QUAST 5.0 is used to evaluate the assembly metrics.

### Benchmarks for scaffolders over different NGS assemblers on *A. thaliana* and *O. sativa*

**Fig. 4:**
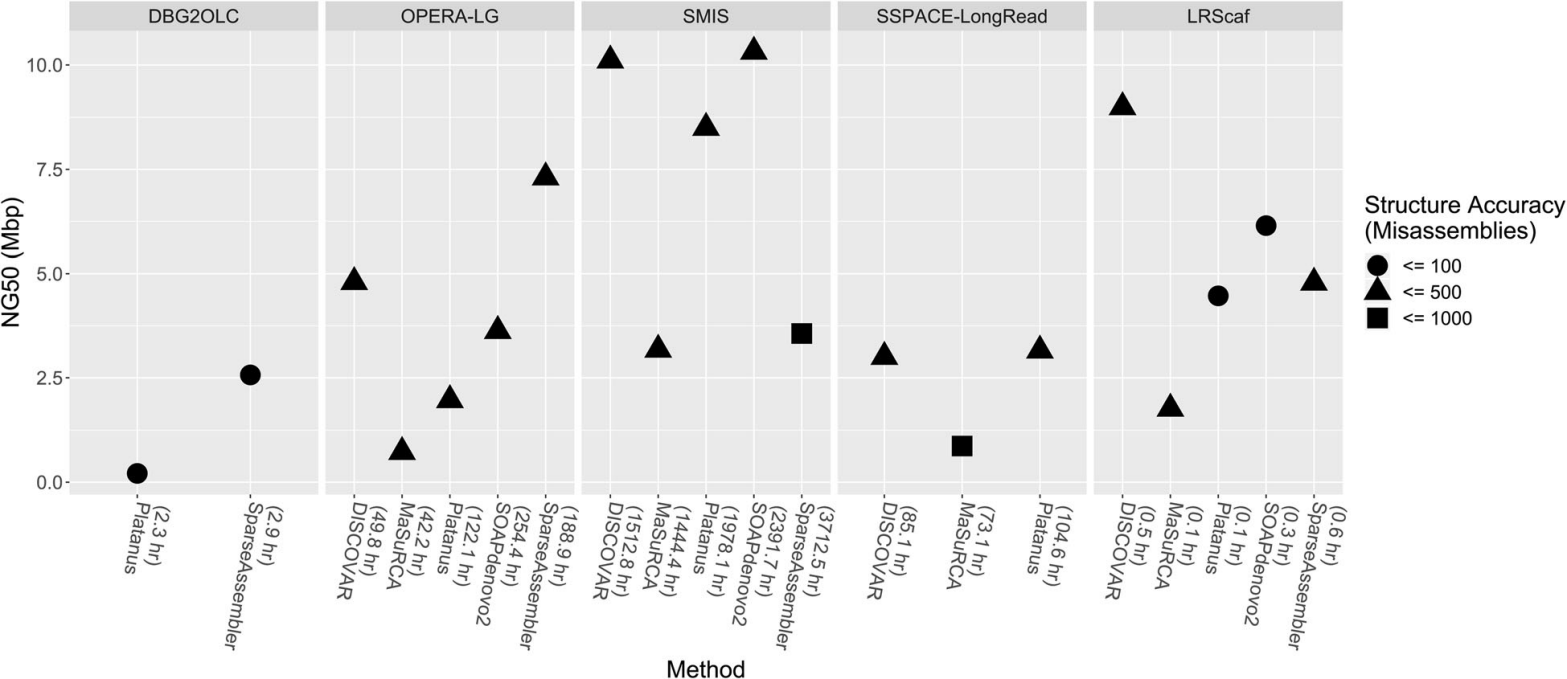
The performances for tested scaffolders over 5 NGS de novo assemblers on *A. thaliana*. The value in parentheses next to NGS de novo assembler is the CPU Time for scaffolder performed on this assembler.

**Fig. 5:**
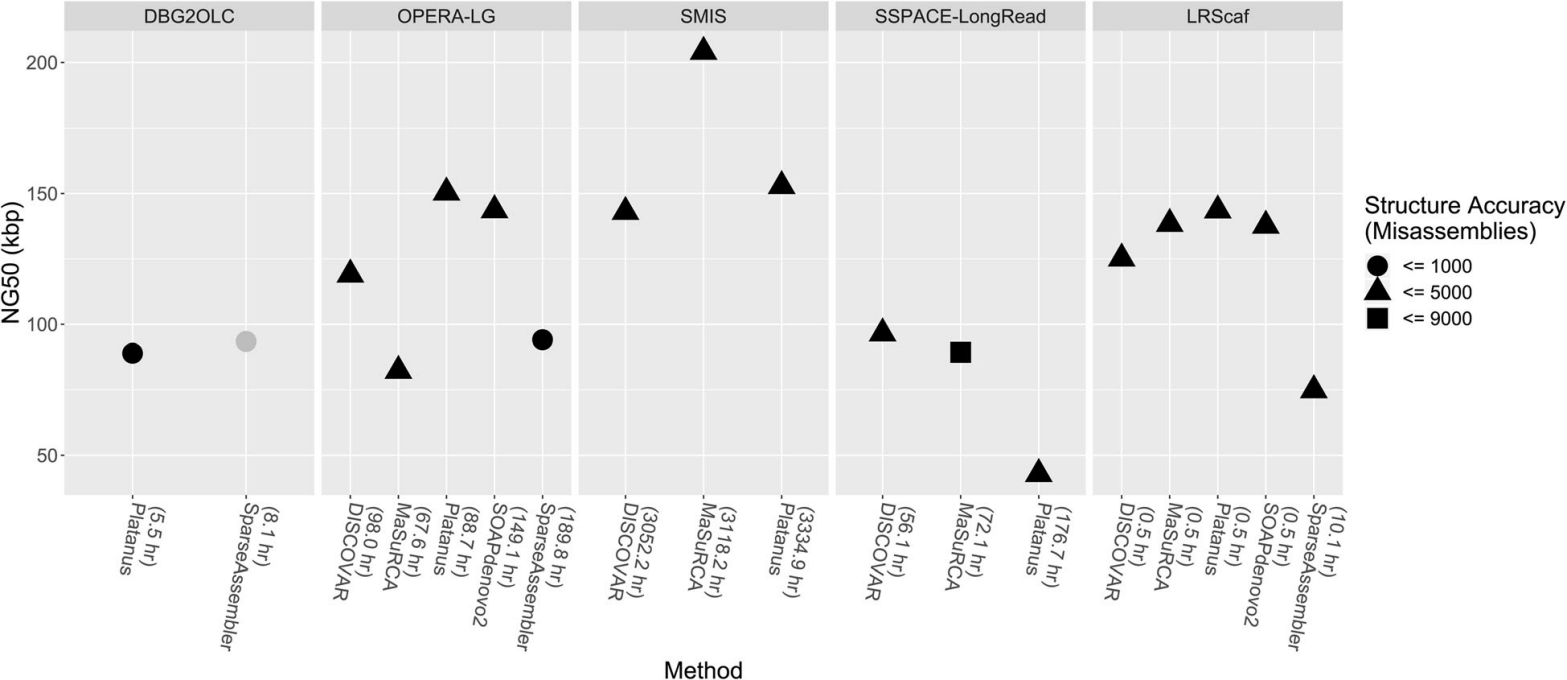
The performances for tested scaffolders over 5 NGS de novo assemblers on *O. sativa*. The value in parentheses next to NGS de novo assembler is the CPU Time for scaffolder performed on this assembler. The number of misassemblies for DBG2OLC on SparseAssembler is not available by QUAST. It denotes in grey.

The NGA50 of draft assemblies for DISCOVAR, MaSuRCA, Platanus, SOAPdenovo2, and SparseAssembler on *A. thaliana* are 314.6 kbp, 356.5 kbp, 143.7 kbp, 227.0 kbp, and 12.7 kbp, respectively (Table 2). As shown in Fig. 4, SMIS and LRScaf perform better on the draft assemblies generated by DISCOVAR, Platanus, and SOAPdenovo2 than the draft assemblies constructed by MaSuRCA and SparseAssembler. OPERA-LG and DBG2OLC yield their best NG50 values on the draft assemblies constructed by SparseAssembler (Additional file 2: Table S6). The benchmarks of SSPACE-LongRead on SOAPdenovo2 and SparseAssembler are excluded in the comparisons because the run time exceeds the one-month time limit. On the *O. sativa* (Fig. 5; Additional file 2: Table S6), the run times of SMIS and SSPACE-LongRead on SOAPdenovo2 and SparseAssembler exceed the one-month time limit. Both of them are excluded from the comparisons. For the draft assemblies generated by Platanus and SOAPdenovo2, OPERA-LG and LRScaf perform better than the other scaffolders. The top-performing scaffolder is SMIS on the draft assemblies generated by MaSuRCA. All of the tested scaffolders perform well on the draft assemblies of DISCOVAR. DBG2OLC yields better performances on SparseAssembler than on Platanus. In summary, these results show that the draft assemblies constructed by DISCOVAR, Platanus, and SOAPdenovo2 are suitable for most scaffolders. Considering on assembly contiguity and structure accuracy, DISCOVAR and SOAPdenovo2 are the recommended NGS de novo assemblers for LRScaf.

### PacBio long read benchmarks

In this study, we use long reads from PacBio datasets for *E. coli*, *S. cerevisiae*, *O. sativa*, *Z. mays*, and *H. sapiens* to assess the performances of seven state-of-the-art scaffolders (i.e., SSPACE-LongRead, LINKS, OPERA-LG, SMIS, npScarf, Unicycler, and DBG2OLC) and LRScaf. For the two small genomes, assembly contiguity obtained from SSPACE-LongRead, SMIS, Unicycler, and LRScaf are generally better than those obtained from LINKS, OPERA-LG, npScarf, and DBG2OLC (Additional file 1 Supplementary Note; Additional file 2: Table S1). Whereas MaSuRCA-Hy generates the best NGA50 value and the longest sequence (187.8 kbp and 2.7 Mbp, respectively) for *O. sativa*, OPERA-LG, and LRScaf yield very similar results (Table 3). The BUSCO results show that OPERA-LG, SMIS, SSPACE-LongRead, and LRScaf have similar quantitative measures. DBG2OLC has 39.9%. OPERA-LG and SMIS fail to run on the draft assemblies of *Z. mays*. LRScaf yields the best NGA50 value and the longest sequence (135.9 kbp and 1.2 Mbp, respectively). The BUSCO assessments for SSPACE-LongRead and LRScaf are 92.7 and 93.4%, respectively. For *H. sapiens* (CHM1), SMIS and SSPACE-LongRead are excluded in the comparisons because they exceed the one-month time limit, and OPERA-LG is excluded because of the lack of NGS data. LRScaf generates the NGA50 value and the longest sequence (9.4 Mbp and 45.0 Mbp, respectively). The assembly contiguity (NG50 and NGA50) is about two times contiguous than that of DBG2OLC. The BUSCO measurement for LRScaf is 94.0%. To summarise, LRScaf yields comparable or superior metrics when compared with other scaffolders on PacBio long reads.

**Table 3:** The performances of tested scaffolders for *O. sativa*, *Z. mays*, and *H. sapiens* using PacBio long reads

Species	Method	Sum	NG50	NGA50	Longest	Mis (#)	BUSCO (Complete)	CPU Time (Hours)	Peak Memory (GB)
*O. sativa*	DBG2OLC (SA)	561.6 Mbp	93.5 kbp	–	488.0 kbp	–	39.9%	8.1	50.6
	MaSuRCA-Hy (MSR)	390.5 Mbp	**248.2 kbp**	**187.8 kbp**	**2.7 Mbp**	1469	**97.8%**	1315.8 (495.1)	353.9
	OPERA-LG (SOAP)	346.1 Mbp	143.6 kbp	98.5 kbp	1.1 Mbp	1346	96.7%	149.1	66.4
	SMIS (MSR)	352.9 Mbp	204.1 kbp	117.5 kbp	1.2 Mbp	1971	97.2%	3118.2	15.7
	SSPACE-LR (DIS)	324.1 Mbp	96.5 kbp	61.1 kbp	1.0 Mbp	3247	97.7%	56.1	**13.6**
	LRScaf (SOAP)	354.6 Mbp	137.7 kbp	102.1 kbp	1.1 Mbp	**1123**	97.9%	**0.5**	28.4
*Z. mays*	SSPACE-LR	2.0 Gbp	48.0 kbp	40.6 kbp	849.5 kbp	**18,407**	92.7%	479.6	**20.1**
	LRScaf	2.4 Gbp	**191.9 kbp**	**135.9 kbp**	**1.2 Mbp**	21,689	**93.4%**	**21.9**	103.8
*H. sapiens* ^(CHM1)^	DBG2OLC^a^	2.8 Gbp	5.5 Mbp	4.5 Mbp	27.3 Mbp	767	93.9%	**–**	–
	LRScaf	2.8 Gbp	**10.4 Mbp**	**9.4 Mbp**	**45.0 Mbp**	**292**	**94.0%**	0.8	20.3

*Note*: The best genomic assembly metrics are highlighted in Bold; Mis: the number of misassemblies; *SA* SparseAssembler, *MSR* MaSuRCA de novo, *SOAP* SOAPdenovo2, *DIS* DISCOVAR de novo, *PLA* Platanus, *SSPACE-LR* SSPACE-LongRead, *MaSuRCA-Hy* MaSuRCA hybrid pipeline; ‘-’: not available; We report two CPU times for MaSuRCA-Hy (the first value is the time for hybrid pipeline and the second value enclosed in the parenthesis is the time for de novo pipeline); OPERA-LG and SMIS are excluded on *Z. mays* because both of them fail to run. SIMS and SSPACE-LongRead are excluded on *H. sapiens* (CHM1) since the run time exceeds the one-month time limit. OPERA-LG is excluded from *H. sapiens* (CHM1) because of the lack of NGS data. ^a^ the assembly is offered by Dr. Chengxi Ye (The developer for DBG2OLC)

### Nanopore long read benchmarks

We use the Nanopore long reads datasets for *E. coli*, *S. cerevisiae*, *A. thaliana*, *S. pennellii*, and *H. sapiens* to assess the performances of the tested scaffolders (Table 4). For the two small genomes, OPERA-LG and Unicycler are excluded from this assessment because of the lack of NGS data. The assembly contiguity obtained from SMIS, npScarf, and LRScaf are generally better than those obtained from LINKS, SSPACE-LongRead, and DBG2OLC (Additional file 1 Supplementary Note; Additional file 2: Table S2). For *A. thaliana*, LRScaf generates the best NGA50 value (4.9 Mbp), and SMIS yields the longest sequence (15.0 Mbp). The BUSCO assessments are similar and over 97.0% for all the scaffolders except for DBG2OLC (25.9%). LRScaf yields the best NGA50 value and the longest sequence for *S. pennellii* (441.3 kbp and 5.4 Mbp, respectively). The BUSCO assessments range from 92.1 to 95.8% except for DBG2OLC (18.9%). SMIS is excluded in the comparison because the run time exceeds the one-month time limit. For *H. sapiens* (NA12878), the run times of SMIS and SSPACE-LongRead exceed the one-month time limit. We do not perform the benchmark for OPERA-LG because of the lack of NGS data. LRScaf generates the NGA50 value and the longest sequence (12.9 Mbp and 64.2 Mbp, respectively), and the BUSCO measurement is 94.9%. Although all scaffolders show some improvements in our experiments, the application of the Nanopore data remains challenging. A recent study showed that the NA12878 genome was assembled with an NG50 value of about 6.5 Mbp using pure 35-fold Nanopore data [6]. Our experiments show that it is possible to improve the assembly contiguity further. Using LRScaf, we obtained an NG50 value of 17.4 Mbp, which is similar to the PacBio human (CHM1) benchmark. To summarise, LRScaf yields either the best or similar assembly metrics using long reads of Nanopore compared with other scaffolders.

**Table 4:** The performances of tested scaffolders for *A. thaliana*, *S. pennellii*, and *H. sapiens* using Nanopore long reads

Species	Method	Sum	NG50	NGA50	Longest	Mis. (#)	BUSCO (Complete)	CPU Time (Hours)	Peak Memory (GB)
*A. thaliana* ^(KBS-Mac-74)^	DBG2OLC (SA)	150.4 Mbp	2.6 Mbp	–	12.2 Mbp	**–**	25.9%	2.9	18.9
	MaSuRCA-Hy (MSR)	123.2 Mbp	2.5 Mb	2.3 Mbp	9.2 Mbp	211	98.3%	145.6 (17.9)	60.3
	OPERA-LG (SA)	116.4 Mbp	7.3 Mbp	2.8 Mbp	14.3 Mbp	**102**	97.2%	188.9	45.9
	SMIS (SOAP)	116.2 Mbp	**10.3 Mbp**	2.4 Mbp	**15.0 Mbp**	180	98.2%	2391.7	26.8
	SSPACE-LR (PLA)	120.6 Mbp	3.2 Mbp	2.2 Mbp	6.8 Mbp	178	**98.4%**	104.6	**9.0**
	LRScaf (DIS)	123.1 Mbp	9.0 Mbp	**4.9 Mbp**	12.4 Mbp	115	98.3%	**0.5**	21.4
*S. pennellii*	DBG2OLC (SA)	1.5 Gbp	243.7 kbp	–	1.7 Mbp	**–**	18.9%	27.0	21.6
	MaSuRCA-Hy (MSR)	950.4 Mbp	331.1 kbp	159.6 kbp	3.1 Mbp	17,957	95.6%	1389.1 (202.8)	239.7
	OPERA-LG (DIS)	952.0 Mbp	730.0 kbp	280.1 kbp	3.5 Mbp	11,404	92.1%	286.6	25.4
	SSPACE-LR (DIS)	871.1 Mbp	82.9 kbp	69.5 kbp	1.3 Mbp	6796	94.7%	650.0	**13.1**
	LRScaf (DIS)	952.8 Mbp	**794.4 kbp**	**441.3 kbp**	**5.4 Mbp**	**5150**	95.8%	**3.1**	36.8
*H. sapiens* ^(NA12878)^	LRScaf	2.9 Gbp	**17.4 Mbp**	**12.9 Mbp**	**64.2 Mbp**	**785**	**94.9%**	**2.1**	**62.6**

*Note*: The best genomic assembly metrics are highlighted in Bold; *Mis*. the number of misassemblies, *SA* SparseAssembler, *MSR* MaSuRCA de novo, *SOAP* SOAPdenovo2, *DIS* DISCOVAR de novo, *PLA* Platanus, *SSPACE-LR* SSPACE-LongRead, *MaSuRCA-Hy* MaSuRCA hybrid pipeline; ‘-’: not available; We report two CPU times for MaSuRCA-Hy (the first value is the time for hybrid pipeline and the second value enclosed in the parenthesis is the time for de novo pipeline); SMIS is excluded on *S. pennellii* because the run time exceeds the one-month time limit. SIMS and SSPACE-LongRead are excluded on *H. sapiens* (NA12878) because the run time exceeds the one-month time limit. OPERA-LG is excluded from *H. sapiens* (NA12878) because of the lack of NGS data

### Computational performance and accuracy analysis

Assembly metrics are undoubtedly the most concerning matters to biologists and bioinformaticians. Nevertheless, from a practical point of view, the run time limits software applications. As evident from our experiments, the run time and the memory usage for scaffolding procedures become significant concerns for large and complex genomes. LRScaf is the fastest scaffolder on the benchmarks. For the PacBio dataset of *O. sativa*, LRScaf reduces the CPU time more than 6700 times compared with SMIS. MaSuRCA-Hy produces the best assembly contiguity. However, its run time is 1600 times longer than LRScaf. For *Z. mays*, LRScaf is 20 times faster than SSPACE-LongRead. On the Nanopore dataset, LRScaf reduces the CPU time more than 4700 times compared with SMIS for *A. thaliana* and 380 times compared with MaSuRCA-Hy for *S. pennellii*. Our experiments show that the memory usage of LRScaf is practical even for large and complex genomes because the peak RAM usage remains below 30.0 GB on the CHM1 PacBio dataset and 70.0 GB on the NA12878 Nanopore dataset respectively.

Reducing the number of misassemblies is important because misassemblies are likely to be misinterpreted as real genetic variations [39, 40]. For the PacBio dataset, LRScaf and SSPACE-LongRead yield the fewest number of misassemblies on *O. sativa* and *Z. mays*, respectively. For the Nanopore dataset, OPERA-LG has the best assembly accuracy on *A. thaliana,* and LRScaf yields the fewest number of misassemblies on *S. pennellii*. LRScaf yields a relatively low number of misassemblies in most of the studied cases. We have no record on the number of misassemblies on *H. sapiens* for the other scaffolders because all of them fail to scaffold the draft assemblies. In summary, LRScaf introduces a new strategy for keeping valid alignments and produces only a moderate number of misassemblies.

## Conclusions

In this work, we present a novel algorithm (LRScaf, see Additional file 3) for scaffolding draft assemblies using noisy TGS long reads and compare our algorithm with published methods. The majority of the draft assemblies constructed using NGS data are fragmented and influenced by repeats. To improve the contiguity of draft assemblies using TGS long reads, there are two strategies on the scaffolding step: 1) simulating mate-pair information (e.g., OPERA-LG and Fast-SG [41]), and 2) using the full-length long reads information (e.g., SSPACE-LongRead and LRScaf). Both strategies could improve the contiguity of draft assemblies. The first strategy could build the scaffolding graph from either short or long reads and have sufficient and convenient linking information from a range of synthetic insert-size mate-pair libraries as well as for the NGS standalone scaffolder reuse. The run time for processing the synthetic linking information and the impact of base accuracy are significant concerns. For the second strategy, it could do the scaffolding step in a speedy and memory-efficient way. Considering only the linking information from long reads would neglect the linking information of paired reads library, whereas integrating paired reads information could improve assembly accuracy. We successfully use long reads to improve draft assemblies over different NGS de novo assemblers. Basing on assembly contiguity and accuracy, DISCOVAR and SOAPdenovo2 are the recommended NGS de novo assemblers for LRScaf.

We propose a new strategy to filter out inaccurate alignments so that these false alignments do not propagate through the scaffolding process. For the assessments on PacBio and Nanopore long-read datasets covering seven organisms, our method shows significant improvements over state-of-the-art scaffolders. The primary benefits of LRScaf are significant reductions in run time and memory usage. These benefits are especially crucial for large and complex genomes. As studied genomes keep getting bigger and more complex, the run time and memory usage of scaffolding software become increasingly essential to biologists and bioinformaticians. Our method is designed to reduce run time and memory usage. Thus, LRScaf is computationally more efficient than other scaffolders. Identification of misassembled contigs is also essential because any misassembled sequences are propagated into the next step during biological analysis. Most state-of-the-art scaffolders lack functions for the identification of misassembled contigs. Besides, misassemblies might be introduced during the scaffolding procedure. Consequently, to limit the number of misassembled scaffolds, our method incorporates an effective validation algorithm to reduce the influence of false alignment.

In the past decade, worldwide collaboration has led to several projects aiming at improving the understanding of species biology and evolution. Examples of such projects are the i5k [42], which provides the genomes of 5000 species of insects, and the Bird 10,000 Genomes (B10K) [43]. A substantial fraction of genomes with short contiguity prevent downstream analysis. Our result shows that TGS data is capable of effectively improving draft assemblies, and LRScaf is a valuable tool for cost-effectively improving these draft assemblies.

## Availability and requirements

Project name: LRScaf.

Project home page: https://github.com/shingocat/lrscaf

Operating system(s): Platform independent.

Programming language: Java.

Other requirements: Java 1.8 or higher.

License: GNU GPL.

Any restrictions to use by non-academics: license needed.

## Supplementary information

**Additional file 1. **Supplementary Note. The additional comparisons and information are included in the Supplementary Note: 1) the assessment for two small genomes on PacBio and Nanopore long reads; 2) the performances of the tested scaffolders over the different depths and medians of long reads on *E. coli* and *H. sapiens*; 3) The comparison of CANU and LRScaf (minimap2) on the different coverages of long reads; 4) The benchmarks for DBG2OLC and LRScaf on *E. coli* and *A. thaliana* using DBG2OLC dataset; 5) The computational system and source code of LRScaf; and 6) The details of parameter settings for LRScaf in the study.

**Additional file 2: **Tables S1-S9. Tables S1–S9 for this study: 1) **Table S1.** The performances for *E. coli* and *S. cerevisiae* based on draft assemblies generated by SOAPdenovo2 and SPAdes using 1, 5, 10, and 20 -fold coverages of PacBio long reads; 2) **Table S2.** The performances for *E. coli* and *S. cerevisiae* based on draft assemblies referred to LINKS on Nanopore long reads; 3) **Table S3.** The performances of scaffolders tested for *H. sapiens* using 1, 5, 15, and 35 -fold coverages of Nanopore long reads; 4) **Table S4.** The performances of all scaffolders tested on different median read lengths (9, 18, and 26 kbp) of 10-fold coverage using PacBio long reads for *E. coli*; 5) **Table S5.** The performances of CANU and LRScaf (minimap2) for *E. coli* on 5, 10, 20, and 30 -fold coverages using Nanopore long reads; 6) **Table S6.** The performances for scaffolder tested on DISCOVAR, MaSuRCA, Platanus, SOAPDenovo2, and SparseAssembler on *A. thaliana*, *O. sativa*, *S. pennellii*, *Z. mays*, and *H. sapiens*; 7) **Table S7.** The BUSCO measurements for scaffolder tested on DISCOVAR, MaSuRCA, Platanus, SOAPDenovo2, and SparseAssembler on *A. thaliana*, *O. sativa*, *S. pennellii*, *Z. mays*, and *H. sapiens*; 8) **Table S8.** The parameter settings of LRScaf on *E. coli*, *S. cerevisiae*, *A. thaliana*, *O. sativa*, *S. pennellii*, *Z. mays*, and *H. sapiens*; and 9) **Table S9.** The performances for DBG2OLC and LRScaf on *E. coli* and *A. thaliana*.

**Additional file 3.** LRScaf (1.1.7). The latest version for LRScaf includes source codes and executable files.

## Data Availability

Sequence data that support the findings of this study have been listed in Table 1. All results generated or analysed during this study are included in this published article.

## Abbreviations

BLASR: Basic Local Alignment with Successive Refinement
LRScaf: Long Reads Scaffolder
NGS: Next Generation Sequencing
SMRT: Single-Molecule, Real-Time
TGS: Third Generation Sequencing

## References

[1] Miller JR, Koren S, Sutton G (2010). Assembly algorithms for next-generation sequencing data. Genomics..

[2] Gnerre S, MacCallum I, Przybylski D, Ribeiro FJ, Burton JN, Walker BJ (2011). High-quality draft assemblies of mammalian genomes from massively parallel sequence data. Proc Natl Acad Sci.

[3] Huson DH, Reinert K, Myers EW (2002). The greedy path-merging algorithm for contig scaffolding. J ACM.

[4] Pop M, Kosack DS, Salzberg SL (2004). Hierarchical scaffolding with Bambus. Genome Res.

[5] Eid J, Fehr A, Gray J, Luong K, Lyle J, Otto G (2009). Real-time DNA sequencing from single polymerase molecules. Science..

[6] Jain M, Koren S, Miga KH, Quick J, Rand AC, Sasani TA (2018). Nanopore sequencing and assembly of a human genome with ultra-long reads. Nat Biotechnol.

[7] Ghurye J, Pop M (2019). Modern technologies and algorithms for scaffolding assembled genomes. PLoS Comput Biol.

[8] Bickhart DM, Rosen BD, Koren S, Sayre BL, Hastie AR, Chan S (2017). Single-molecule sequencing and chromatin conformation capture enable de novo reference assembly of the domestic goat genome. Nat Genet.

[9] Dudchenko O, Batra SS, Omer AD, Nyquist SK, Hoeger M, Durand NC (2017). De novo assembly of the Aedes aegypti genome using hi-C yields chromosome-length scaffolds. Science.

[10] Chin CS, Alexander DH, Marks P, Klammer AA, Drake J, Heiner C (2013). Nonhybrid, finished microbial genome assemblies from long-read SMRT sequencing data. Nat Methods.

[11] Burton JN, Adey A, Patwardhan RP, Qiu R, Kitzman JO, Shendure J (2013). Chromosome-scale scaffolding of de novo genome assemblies based on chromatin interactions. Nat Biotechnol.

[12] Chen Lei, Qiu Qiang, Jiang Yu, Wang Kun, Lin Zeshan, Li Zhipeng, Bibi Faysal, Yang Yongzhi, Wang Jinhuan, Nie Wenhui, Su Weiting, Liu Guichun, Li Qiye, Fu Weiwei, Pan Xiangyu, Liu Chang, Yang Jie, Zhang Chenzhou, Yin Yuan, Wang Yu, Zhao Yue, Zhang Chen, Wang Zhongkai, Qin Yanli, Liu Wei, Wang Bao, Ren Yandong, Zhang Ru, Zeng Yan, da Fonseca Rute R., Wei Bin, Li Ran, Wan Wenting, Zhao Ruoping, Zhu Wenbo, Wang Yutao, Duan Shengchang, Gao Yun, Zhang Yong E., Chen Chunyan, Hvilsom Christina, Epps Clinton W., Chemnick Leona G., Dong Yang, Mirarab Siavash, Siegismund Hans Redlef, Ryder Oliver A., Gilbert M. Thomas P., Lewin Harris A., Zhang Guojie, Heller Rasmus, Wang Wen (2019). Large-scale ruminant genome sequencing provides insights into their evolution and distinct traits. Science.

[13] English AC, Richards S, Han Y, Wang M, Vee V, Qu J (2012). Mind the gap: upgrading genomes with Pacific biosciences RS Long-read sequencing technology. PLoS One.

[14] Chateau A, Giroudeau R (2015). A complexity and approximation framework for the maximization scaffolding problem. Theor Comput Sci.

[15] Salmela L, Mäkinen V, Välimäki N, Ylinen J, Ukkonen E (2011). Fast scaffolding with small independent mixed integer programs. Bioinformatics..

[16] Sequences HP (2011). Opera : reconstructing optimal genomic scaffolds. J Comput Biol.

[17] Donmez N, Brudno M (2013). SCARPA: scaffolding reads with practical algorithms. Bioinformatics..

[18] Dayarian A, Michael TP, Sengupta AM (2010). SOPRA: scaffolding algorithm for paired reads via statistical optimization. BMC Bioinformatics.

[19] Boetzer M, Henkel CV, Jansen HJ, Butler D, Pirovano W (2011). Scaffolding pre-assembled contigs using SSPACE. Bioinformatics..

[20] Sahlin K, Vezzi F, Nystedt B, Lundeberg J, Arvestad L (2014). BESST - efficient scaffolding of large fragmented assemblies. BMC Bioinformatics.

[21] Luo J, Wang J, Zhang Z, Li M, Wu FX (2017). BOSS: a novel scaffolding algorithm based on an optimized scaffold graph. Bioinformatics..

[22] Hunt M, Newbold C, Berriman M, Otto TD (2014). A comprehensive evaluation of assembly scaffolding tools. Genome Biol.

[23] Bashir A, Klammer AA, Robins WP, Chin C-S, Webster D, Paxinos E (2012). A hybrid approach for the automated finishing of bacterial genomes. Nat Biotechnol.

[24] Chaisson MJ, Tesler G (2012). Mapping single molecule sequencing reads using basic local alignment with successive refinement (BLASR): application and theory. BMC Bioinformatics.

[25] Li H (2018). Minimap2: pairwise alignment for nucleotide sequences. Bioinformatics.

[26] Boetzer M, Pirovano W (2014). SSPACE-LongRead: scaffolding bacterial draft genomes using long read sequence information. BMC Bioinformatics.

[27] Warren RL, Yang C, Vandervalk BP, Behsaz B, Lagman A, Jones SJM (2015). LINKS: scalable, alignment-free scaffolding of draft genomes with long reads. Gigascience.

[28] Gao S, Bertrand D, Chia BKH, Nagarajan N (2016). OPERA-LG: efficient and exact scaffolding of large, repeat-rich eukaryotic genomes with performance guarantees. Genome Biol.

[29] Cao MD, Nguyen SH, Ganesamoorthy D, Elliott AG, Cooper MA, Coin LJM (2017). Scaffolding and completing genome assemblies in real-time with nanopore sequencing. Nat Commun.

[30] Ye C, Hill CM, Wu S, Ruan J, Ma Z (2016). DBG2OLC: efficient assembly of large genomes using long erroneous reads of the third generation sequencing technologies. Sci Rep.

[31] Wick RR, Judd LM, Gorrie CL, Holt KE (2017). Unicycler: resolving bacterial genome assemblies from short and long sequencing reads. PLoS Comput Biol.

[32] Luo R, Liu B, Xie Y, Li Z, Huang W, Yuan J (2012). SOAPdenovo2: an empirically improved memory efficient short-read de novo assembler. Gigascience.

[33] Bankevich A, Nurk S, Antipov D, Gurevich AA, Dvorkin M, Kulikov AS (2012). SPAdes: a new genome assembly algorithm and its applications to single-cell sequencing. J Comput Biol.

[34] Weisenfeld NI, Yin S, Sharpe T, Lau B, Hegarty R, Holmes L (2014). Comprehensive variation discovery in single human genomes. Nat Genet.

[35] Zimin AV, Marçais G, Puiu D, Roberts M, Salzberg SL, Yorke JA (2013). The MaSuRCA genome assembler. Bioinformatics..

[36] Kajitani R, Toshimoto K, Noguchi H, Toyoda A, Ogura Y, Okuno M (2014). Efficient de novo assembly of highly heterozygous genomes from whole-genome shotgun short reads. Genome Res.

[37] Ye C, Ma ZS, Cannon CH, Pop M, Yu DW (2012). Exploiting sparseness in de novo genome assembly. BMC Bioinformatics.

[38] Chakraborty M, Baldwin-Brown JG, Long AD, Emerson JJ (2016). Contiguous and accurate de novo assembly of metazoan genomes with modest long read coverage. Nucleic Acids Res.

[39] Salzberg SL, Yorke JA (2005). Beware of mis-assembled genomes. Bioinformatics..

[40] Muggli MD, Puglisi SJ, Ronen R, Boucher C (2015). Misassembly detection using paired-end sequence reads and optical mapping data. Bioinformatics..

[41] Di Genova A, Ruz GA, Sagot M-F, Maass A (2018). Fast-SG: an alignment-free algorithm for hybrid assembly. Gigascience.

[42] Robinson GE, Hackett KJ, Purcell-Miramontes M, Brown SJ, Evans JD, Goldsmith MR (2011). Creating a buzz about insect genomes. Science.

[43] Zhang G (2015). Genomics: bird sequencing project takes off. Nature.

