# Supplementary material for: LRScaf: improving draft genomes using long noisy reads
Source: BMC Genomics. 2019 Dec 9;20:955. doi: 10.1186/s12864-019-6337-2 (PMC6902338; doi:10.1186/s12864-019-6337-2)
Supplement: Supplementary file 1 — Additional file 1. Supplementary Note. The additional comparisons and information are included in the Supplementary Note: 1) the assessment for two small genomes on PacBio and Nanopore long reads; 2) the performances of the tested scaffolders over the different depths and medians of long reads on E. coli and H. sapiens; 3) The comparison of CANU and LRScaf (minimap2) on the different coverages of long reads; 4) The benchmarks for DBG2OLC and LRScaf on E. coli and A. thaliana using DBG2OLC dataset; 5) The computational system and source code of LRScaf; and 6) The details of parameter settings for LRScaf in the study. [file 12864_2019_6337_MOESM1_ESM.docx]

Supplementary Note

# The performances for two small genomes on PacBio long reads

We assessed the performances of all scaffolders on PacBio datasets for *E. coli* and *S. cerevisiae* (Table S1). On the draft assemblies constructed by SOAPdenovo2 on 20-fold coverage, SSPACE-LongRead, SMIS, and LRScaf have the best assembly contiguity and accuracy scaffolds. Whereas SSPACE-LongRead, Unicycler, and LRScaf yield the best assembly contiguity on 20-fold coverage with SPAdes draft assembly, LINKS, OPERA-LG, SMIS, npScarf, and DBG2OLC fail to do that. Besides, Unicycler yields the fewest number of misassemblies on 1, 5, 10, and 20 -fold coverages*.* On the draft assembly of *S. cerevisiae* generated by SOAPdenovo2 on 20-fold coverage, SSPACE-LongRead yields the best assembly contiguity (NG50). SSPACE-LongRead and LRScaf generate the longest sequence. On the draft assemblies generated by SPAdes, the npScarf method yields the best NG50 value (665.8 Kb) on 20-fold coverage. SSPACE-LongRead, LINKS, OPERA-LG, npScarf, and LRScaf yield the longest sequence (1.0 Mb). In summary, Unicycler and LRScaf are capable of constructing accuracy assemblies than other scaffolders on small genomes.

# The performances for two small genomes on Nanopore long reads

The assembly metrics of SMIS and LRScaf for *E. coli* are better than these of npScarf (Table S2). Nevertheless, the assembly contiguity of npScarf for *S. cerevisiae* is better than that of SMIS and LRScaf. For *E. coli*, LRScaf yields the best assembly contiguity using the FULL dataset. Besides, SMIS and LRScaf generate the fewest number of misassemblies. SMIS yields the best assembly contiguity using the ALL and RAW datasets. For *S. cerevisiae*, the npScarf method yields the best NG50 value and the longest sequence where SMIS yields the best NA50 value and the fewest number of misassemblies using the NANOCORR datasets. The npScarf produces the best assembly contiguity and LRScaf with minimap2 generates the fewest number of misassemblies using RAW Nanopore datasets. The npScarf method yields the maximum number of misassemblies in all datasets for *E. coli* and *S. cerevisiae*.

# The performances for tested scaffolders on the different depth of long reads

The requirements of computational resources for all the scaffolders were practical on small genomes (Table S1). Nevertheless, these requirements were intensive for scaffolding human genomes (Table S3), *e.g.* the run time for SMIS and SSPACE-LongRead were over one-month on 1-fold coverage, the peak RAM for LINKS exceeded the memory capacity of system (1 Tb) on 1-fold coverage, and the assembly size for DBG2OLC was much smaller than the expected human genome size.

We have assessed the performances of scaffolders tested on different depths (1, 5, 10, and 20 -fold) for two small genomes (*E. coli* and *S. cerevisiae*) using the PacBio datasets (Table S1). In summary, the assembly contiguity improves as the coverage of long reads increases. The assembly contiguity is significantly increased from 1-fold to 5-fold and from 5-fold to 10-fold coverage, whereas this growth rate of assembly contiguity is relatively flat from 10-fold to 20-fold coverage. For *E. coli*, Unicycler yields a nearly complete genome (NG50: 4.4 Mb) using 5-fold coverage. SSPACE-LongRead, Unicycler, and LRScaf yield similar assembly contiguity using 10-fold coverage. On 20-fold coverage, SSPACE-LongRead, Unicycler, and LRScaf construct the best assembly contiguity (NG50: 4.6 Mb). On *S. cerevisiae*, the assembly contiguity for most scaffolders is similar in 1 and 5 -fold coverages. SSPACE-LongRead and npScarf yield close assembly contiguity in 10-fold coverage. Moreover, npScarf yields the best NG50 value (665.8 Kb) using 20-fold coverage.

We extracted 1, 5, and 15 -fold coverages from the NA12878 Nanopore dataset and used four different coverages, *i.e.* 1, 5, 15, and 35 -fold, to assess the performance for all the scaffolders tested on large genomes (Table S3). As the coverage of reads grows, the assembly contiguity is improved. The assembly contiguity for npScarf is slightly better than LRScaf on 1, 5, and 15 -fold coverages. Nevertheless, its run time and the number of misassemblies are worse than LRScaf. The run time for npScarf exceeded the one-month time limit on 35-fold coverage where LRScaf with minimap2 improves the assembly contiguity NG50 value from 115.7 Kb to 17.4 Mb. The draft assemblies constructed by DISCOVAR might be the reason for the assembly-genome size generated by DBG2OLC which is recommended to use SparseAssembler as its NGS *de novo* assembler was much smaller than the expected human genome size.

# The performances for tested scaffolders on different median length of long reads

We assessed the performances of all scaffolders tested on different median length (9, 18, and 26 Kb) of reads at a pre-defined coverage (10-fold) for *E. coli* (Table S4). We chose 26 Kb instead of 28 Kb, because of lacking read coverage in sampling 28 Kb tier. As the median length of long reads increases, the assembly contiguity is improved. The assembly contiguity is significantly increased from 9 Kb to 18 Kb, whereas the growth rate of assembly contiguity is relatively flat from 18 Kb to 26 Kb. Unicycler, npScarf, SSPACE-LongRead, and LRScaf (minimap2) yield the best assembly contiguity (NG50). SSPACE-LongRead, SMIS, npScarf, Unicycler, and LRScaf construct a complete genome (NG50 = 4.6 Mb) on the median length of 18 and 26 Kb datasets. Besides, Unicycler yields the fewest number of misassemblies in all cases.

# The comparison between CANU and LRScaf (minimap2)

We assessed the performances of CANU and LRScaf on 5, 10, 20, 30, and 60 -fold coverages for *E. coli* using PacBio dataset (Table S5). As the coverage of long reads increases, the assembly contiguity for these two algorithms is improved. Because the genome for *E. coli* is small and not complicated, LRScaf is capable of constructing a complete genome (NG50 = 4.6 Mb) on 20-fold coverage where CANU needs 30-fold coverage to do that. On the assessments of 5, 10, and 20 -fold coverages, LRScaf yields better assembly contiguity and more accurate assembly than CANU. These results are as expected because the recommended minimum coverage of CANU is between 30 and 60 -fold. CANU is a pure TGS *de novo* assembler and not designed for the low coverage. Nevertheless, LRScaf is a standalone scaffolder aiming at improving draft assemblies with low-depth TGS data (about 20-fold coverage), especially on large genomes for which the computational cost and budget are significant constraints. On the 60-fold coverage, the polished assembly of CANU outperformed than the LRScaf. The number of indels is the significant issue on affecting the assembly accuracy on low coverage (5, 10, and 20 -fold)

The PacBio dataset for *E. coli* is on SRX669475 and SRX533603. We random subsample 5, 10, 20, 30, and 60 coverage. We used these sub-datasets to assess the performances of CANU and LRScaf. SOAPdenovo2 constructs the draft assembly.

# The benchmarks for DBG2OLC and LRScaf on *E. coli*, *A. thaliana*, and *H. sapiens* using DBG2OLC dataset

We assessed the performances for DBG2OLC and LRScaf on *E. coli*, *A. thaliana*, and *H. sapiens* (Supplementary Table 9). Both of scaffolders could improve the assembly contiguity. The BUSCO measurements for DBG2OLC are worse than the draft assemblies. The BUSCO measurements for LRScaf significantly improve after scaffolding step. On *E. coli*, both of scaffolds could improve the draft assembly closed to genome size with NG50 value 4.6 Mbp. On *A. thaliana*, the NG50 value for DBG2OLC is about two times longer than that of LRScaf. The assembly contiguity (NG50 and NGA50) for LRScaf is about two times longer than that of DBG2OLC on *H. sapiens*. The run time for LRScaf is faster two times than DBG2OLC on *E. coli*, however, slower two times than DBG2OLC on *A. thaliana*. The memory usage for LRScaf is superior or equal to that for DBG2OLC.

# The computational system information and source code

The draft assembly’s construction and the scaffolding procedures for two small genomes and *H. sapiens* were performed on a 1 Tb memory Linux machine with 48 CPUs incorporating Hyper-threading technology. The scaffolding procedures for *A. thaliana*, *O. sativa*, *S. pennellii*, and *Z. mays* were performed on a cluster with three nodes. Each node is 256 Gb memory capacity and 24 CPUs. The memory usage of construction for the draft assemblies of *Z. mays* exceeded our maximum memory capacity (1 Tb). Hence, we used the draft assemblies GCA_000005005.5, GCF_000306695.2, and GCA_001517065.4 for *Z. mays* and *H. sapiens* (CHM1 and NA12878), respectively. All the methods are set to 8 threads if it supports multi-threads.

LRScaf is written in Java™ and is capable of running on all platforms including Linux, Windows, and Mac if Java Running Environment (JRE) installed. The source code is available on GitHub (https://github.com/shingocat/lrscaf). We provide a packaged jar file which could be used straight out of the box and the compilation steps for advanced users.

# The parameters settings for LRScaf in the benchmark

The values of the parameter for LRScaf is adjusted according to three main factors: the contiguity of draft assembly, the coverage of long reads, and the TGS mapper (BLASR or minimp2). The most significant difference is the identity parameter for LRScaf with different TGS mapper. The value of identity parameter for LRScaf with BLASR is over 0.7 in 26 out of 29 cases whereas this value is 0.1 in 25 out of 29 cases on LRScaf with minimap2. The value of min_contig_length is adjusted basing on the assembly contiguity of draft assembly. The value of mini_supported_links is adjusted basing on the coverage of long reads.
